# Various Expressions of *PIK3C2A* and *TXNIP* Genes and Their Potential Role as Independent Risk Factors for Chronic Stable Angina and Acute Coronary Syndrome

**DOI:** 10.3390/biom13020302

**Published:** 2023-02-06

**Authors:** Shimaa E. Soliman, Mai A. H. Abouelenin, Neven I. Samy, Marwa M. Omar, Abeer A. Alrefai

**Affiliations:** 1 Medical Biochemistry and Molecular Biology Department, Faculty of Medicine, Menoufia University, Shebin el Kom 32511, Egypt; 2 Medical Biochemistry Unit, Department of Pathology, College of Medicine, Qassim University, Buraydah 51452, Saudi Arabia; 3 Cardiovascular Department, Faculty of Medicine, Menoufia University, Shebin el Kom 32511, Egypt; 4 Clinical Pathology Department, Faculty of Medicine, Menoufia University, Shebin el Kom 32511, Egypt; 5 Biochemistry Department, Faculty of Medicine, Umm Al-Qura University, Makkah 21955, Saudi Arabia

**Keywords:** *PIK3C2A*, *TXNIP*, CSA, ACS, real-time PCR

## Abstract

Background and Aim: Genetic factors play a significant role in the onset and progression of coronary artery disease (CAD). *PIK3C2A* may contribute to the development of acute coronary syndrome (ACS) by affecting blood glucose levels and oxidative stress. The expression levels of *TXNIP* were significantly higher in patients with unstable angina pectoris. However, the situation is different in ACS. In the current study, we aim to investigate the role of *PIK3C2A* and *TXNIP* as independent risk factors for chronic stable angina (CSA) and ACS. Subjects and Methods: This study involved 215 subjects (60 patients with CSA, 55 patients with ACS, and 100 controls). All subjects were exposed for assaying gene expressions of *PIK3C2A* and *TXNIP* by quantitative real-time polymerase chain reaction. Results: It was found that *TXNIP* was upregulated, whereas *PIK3C2A* was downregulated in patients with CAD compared to the control group. *PIK3C2A* was significantly downregulated in patients with ACS compared to that in patients with CSA (*p* < 0.001), but *TXNIP* was not (*p* = 0.7). *TXNIP* was significantly upregulated in STEMI-ACS patients compared to CSA (*p* = 0.045) and NSTEMI ACS (*p* = 0.046), among non-diabetic (*p* = 0.023) smokers (*p* = 0.036) with hypertension (*p* = 0.005) and hypercholesterolemia (*p* = 0.001). ROC (receiver operating characteristic) curve analysis revealed that *PIK3C2A* (0.981; *p* < 0.001; 98.18) was the most sensitive mRNA for discriminating ACS from control, followed by *TXNIP* (0.775; *p* < 0.001; 70.91). However, for discriminating ACS from CSA combined mRNAs, (*PIK3C2A + TXNIP*) (0.893; *p* < 0.001; 98.18) and *PIK3C2A* (0.892; *p* < 0.001; 81.82) are promising biomarkers. On the other hand, the most sensitive mRNA for differentiating CSA from control is mRNAs (*PIK3C2A + TXNIP*) (0.963; *p* < 0.001; 95), then *TXINP* (81.3; *p* < 0.001; 93.33), and finally, *PIK3C2A* (0.782; *p* < 0.001; 81.67). In the multivariate regression model, *PIK3C2A* ((*p* = 0.002), 0.118 (0.031–0.445)) and smoking status ((*p* = 0.034); 0.151 (0.026–0.866)) were independent variables for ACS. Moreover, *PIK3C2A* ((*p* < 0.013); 0.706 (0.614–0.812)), Hb ((*p* = 0.013); 0.525 (0.317–0.871)), and total cholesterol ((*p* = 0.04); 0.865 (0.784–0.955)) were significantly (*p* < 0.05) and independently related to the prognosis of CSA. Furthermore, *PIK3C2A* ((*p* = 0.002), 0.923 (0.877–0.971)), *TXNIP* ((*p* = 0.001); 2.809 (1.558–5.064)) the body weight ((*p* = 0.033); 1.254 (1.018–1.544)) were independently associated with CSA. Conclusions: Our study concluded that the dysregulated mRNA *PIK3C2A* and *TXNIP* gene expressions may be useful in diagnosis of CAD and prediction of ACS development.

## 1. Introduction

Prolonged ischemia of the coronary artery can lead to acute coronary syndrome (ACS) in patients with coronary artery disease (CAD) [1]. Important causes of death in developed countries are known to be CAD and ACS [2]. Risk factors for thrombosis and CVD include unhealthy eating habits, inactivity, dyslipidemia, diabetes mellitus, hypertension, obesity, differences in sex and race, smoking, renal diseases, and familial hypercholesterolemia [3]. The arterial function is an important “risk marker” in CVD; assessment of arterial stiffness can detect the slight modification in the arterial function before the appearance of clinical signs [4]. Prevention of CVD has improved with identification of these classical risk factors [5]. The overall mortality from CVD is still increasing as vascular phenotypes have a high hereditary component [6]. According to population and sibling studies, genetic variables are thought to be responsible for 40–60% of CAD susceptibility [7].

The effect of cellular and molecular levels on the pathological processes causing ACS and the therapeutic methods have been considered in developed treatment techniques established over the last few decades [8]. Nowadays, genetic markers can be measured noninvasively, making it easier to identify a hereditary predisposition to CAD [9]. It is convenient to screen for high-risk individuals very early in life by measuring such indicators. Moreover, genetic markers do not fluctuate as circulating indicators, such as triglycerides or cholesterol, do. Therefore, more timely screening may provide better preventative measures (drug and lifestyle modifications), given that early risk factor management has the potential to delay or prevent the condition [10].

The PI3K (phosphoinositide 3-kinase) gene codes for 1068 amino acids and is located on chromosome 3q26, is 34 kb in size, and consists of 20 exons [11]. *PIK3C2A* is a class II member of lipid kinases of the family of PI3K that catalyzes the phosphorylation of phosphatidylinositol (PI) in the 3-OH position and regulates pathways of signals [12]. It has a regulatory role in platelet function and arterial thrombosis since its structure differs from that of the other class members, and it is expressed in smooth muscle, endothelial cells, and vascular endothelium [13]. Angiogenesis may be affected by *PIK3C2A* and affect the pathophysiology of CAD [14]. The main function of *PIK3C2A* is vesicular trafficking, which is crucial for endothelial cell growth, survival, migration, morphogenesis, and ultimately, angiogenesis [15].

PIK3C2A activation was found to depend on several receptors, including tyrosine kinase receptors, as well as insulin receptor (IR), epidermal growth factor receptor (EGFR), transforming growth factor beta receptor 1 (TGFBR1), vascular endothelial growth factor receptor (VEGFR), as well as G-protein coupled receptors such as sphingosine-1-phosphate receptor 1 (S1PR1) and C-X-C motif chemokine receptor 2 (CXCR2) [16]. According to several studies, thioredoxin-interacting protein (TXNIP) is implicated in various physiologic and pathological processes, including ischemia/reperfusion. An endogenous inhibitor and regulator of thioredoxin (TRX) is the TXNIP protein, also known as vitamin D3-upregulated protein-1 [17]. It belongs to the superfamily of α-arrestin proteins and was first identified as a regulator of oxidative stress through the inhibition of thioredoxin activity [18]. *TXNIP* also plays significant roles in inflammatory reactions and cell death depending on the buildup of reactive oxygen species (ROS) and production of oxidative stress [19].

This study aims to explore the potential roles of *PIK3C2A* and *TXNIP* as biomarkers to predict the risk of CAD involving CSA and ACS. 

## 2. Patients and Methods

### 2.1. Study Design and Subjects

This is a case-control study that was conducted at the Department of Medical Biochemistry and Molecular Biology in collaboration with the Departments of Cardiovascular and Clinical Pathology, Menoufia University Hospitals. This study included 215 subjects categorized into three groups: Group I included 100 subjects with no CAD who had normal coronary angiography; Group II: consisted of 60 patients with CSA who were referred for elective coronary angiography; Group III included 55 patients with ACS (either STEMI = 40 patients or NSTEMI = 15 patients) chosen from the Coronary Care Unit (CCU).

### 2.2. Inclusion and Exclusion Criteria

STEMI (ST-elevation myocardial infarction) was established according to the Fourth Universal Definition of Myocardial Infarction in ESC Guidelines 2018 [20], which is defined as the detection of a rise and/or fall in cardiac troponin with at least one value above the 99th percentile upper reference limit, symptoms of acute myocardial ischemia, and ischemic ECG criteria. Moreover, new ST-elevation at the J-point in two adjacent leads with a cut-point less than 1 mm in all other leads in addition to leads V2–V3 (where the following cut-points are applicable. 2.5 mm in men over 40, 1.5 mm in women of any age, and 2 mm in men under 40). On the other hand, NSTEMI (non ST-elevation myocardial infarction) was defined as the rise and fall of cardiac biomarkers (ideally troponin) with at least one value over the upper 99th percentile reference limit and accompanied by one of the following: the emergence of pathologic Q waves on the ECG, the presence of new ST-segment/T-wave changes (such as ST depression or T-wave inversions), signs of ischemia, imaging evidence of a loss of viable myocardium, or a new regional wall motion abnormality [21]. Patients with heart failure, congenital heart conditions, and associated liver or renal problems were excluded from our groups. All the study patients were subjected to full history taking, including age, gender, and risk factors profile (such as diabetes mellitus, hypertension, smoking, positive family history, and previous myocardial infarction), and both general and local heart assessment were part of the comprehensive clinical evaluation. An electrocardiogram was taken for diagnosis of ACS (rate, rhythm, conduction abnormalities). Moreover, laboratory investigations, including CBC, lipid profile, cardiac troponin, and coronary angiography, were conducted, and standard left and right coronary angiograms with at least two of the best forecasts for each patient were obtained. Each angiogram was evaluated using a set of parameters by two expert interventional cardiologists, who then agreed on the results by identifying the significance of coronary artery lesions (significant coronary stenosis is defined according to the American College of Cardiology/American Heart Association as ≥50% narrowing of the lumen diameter in at least one major coronary artery) if present and the number of vessels diseased [22]. 

### 2.3. Blood Sample Collection and Processing

Seven milliliters (7 mL) of venous blood from each participant was drawn over the first 24 h following ACS and after a 12-h fast. The samples then were processed as follows: 3 mL was added to an EDTA-containing tube for CBC and RNA extraction to process the *PIK3C2A* and *TXNIP* gene expressions utilizing real-time qPCR. For serum isolation, the remaining 4 mL was transferred to a plain tube and centrifuged at 4000 rpm for 10 min.

### 2.4. Biochemical Investigations

The serum was preserved and kept at −80 °C till the lipid profile assessments were made. Total cholesterol (TC), triacylglycerol (TG), and high-density lipoprotein cholesterol (HDLc) levels were measured in all study participants using kits from Spinreact (Girona, Spain) and Human Kit (Wiesbaden, Germany), respectively. Moreover, standard colorimetric procedures were also used to measure these values. The low-density lipoprotein cholesterol (LDLc) value was calculated by the Friedewald equation: LDLc = TC − (TG/5 + HDLc) [23]. The very low-density lipoprotein cholesterol (VLDL-c) value was estimated by the Friedewald equation: (VLDL-c) = TG/5 mg/dL, as TG levels did not exceed 400 mg/dL [23]. Treponin was qualitatively assessed in our subjects. Furthermore, CBC was assessed using Sysmex XN-1000 (Kobe, Japan (19723), BMEgypt Company).

### 2.5. RNA Isolation and Real-Time Quantitative PCR (qRT-PCR)

Blood was processed using the QIAamp RNA Blood Mini Kit to produce total RNA (Qiagen, Hilden, Germany). cDNA was created using RT-PCR (MyTaq™ One-Step RT-PCR Kit). We added 1 μL of enzyme for reverse transcriptase, 4 μL of 5x TransAmp buffer, and 5 μL of RNase-free water to 10 μL of RNA extract. The enzyme for reverse transcriptase was inhibited using the Applied Biosystems 2720 Thermal Cycler (Bioline, London, UK), which was used at 25 °C for a single cycle of 10 min, at 42 °C for 15 min, and finally, at 85 °C for 5 min. The created cDNA was stored at 20 °C.

**Figure 1:**
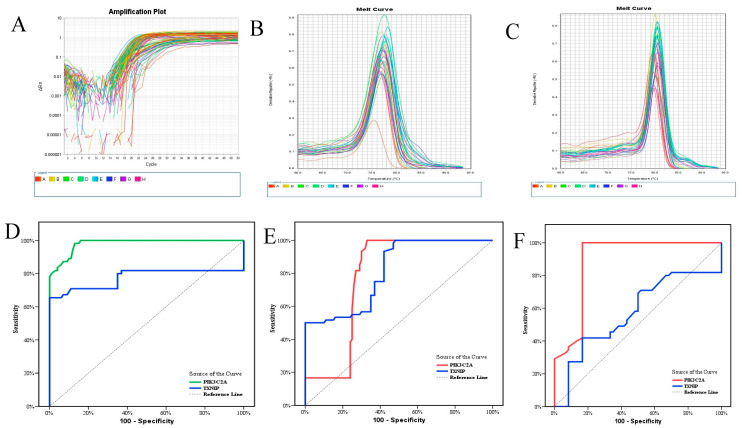
(**A**) Amplification plot of PIK3C2A& TXNIP (Rn vs. Cycle) (**B**) Melt Curve of PIK3C2A (**C**) Melt Curve of TXNIP (**D**) ROC curve for PIK3C2A and TXNIP to diagnose Acute Coronary Syndrome (ACS) from control (**E**) ROC curve for PIK3C2A and TXNIP to diagnose Chronic Stable Angina (CSA) from control (**F**) ROC curve for PIK3C2A and TXNIP to diagnose Acute Coronary Syndrome (ACS).

The Quanti Tect SYBR Green PCR Kit with ready-made Quanti Tect Primer Assay (Qiagen) was used to conduct SYBR green-based qRT-PCR using the cDNA. The following primers were used to assess the amounts of mRNA for *PIK3C2A* and *TXNIP: PIK3C2A* Sense, 5′-CTCAGCTTGCAAAAGCCCAG-3′ and Antisense, 5′-CTGGGTTTGTGCGGTGATTG-3′; *TXNIP* gene Sense, 5′-GCCACACTTACCTTGCCAAT-3′ and Antisense, 5′-TTGGATCCAGGAACGCTAAC-3′. Primers for human glyceraldehyde-3-phosphate dehydrogenase (*GAPDH*) were as follows: Sense, 5′-TCCATGACAACTTTGGCATCGTGG-3′ and Antisense, 5′-GTTGCTGTTGAAGTCACAGGAGAC-3′. We mixed 5 μL of the cDNA with 10 μL of 2 x SYBR^®^ Low ROX Master Mix, 1 μL of each primer, and 3 μL of RNase-free water to create a 20 μL mixture. The reaction was conducted in 45 cycles with 30 s of denaturation at 94 °C, 30 s of annealing at 55 °C, and 30 s of extension at 72 °C. Version 2.0.1 of the Applied Biosystems 7500 software was used to analyze the data. The results were interpreted using the comparative CT method (2−ΔΔCt), and the relative quantification (RQ) method was used to quantify the definite gene, optimize the results to *GAPDH* (an endogenous reference gene), and then compare these results to control (Figure 1A). To verify the specificity and identity of the PCR product and to prevent primer dimers, a melting curve analysis was carried out in each run (Figure 1B,C).

Sample Size Estimation: Based on a review of the previous literature [24], the expression of *TXNIP* in CAD was significantly higher than that in controls (*p* = 0.05). To detect this significant difference, we used an unpaired *t* test with a power of 80%, a confidence level of 95%, and a margin of error of 5%. This study included 215 participants.

Statistical Analysis: Data analysis was performed using the IBM SPSS software package version 20.0 (IBM Corp., Armonk, NY, USA). Using the Chi-square test, we compared the categorical variables between groups (Fisher’s exact test or Monte Carlo). For quantitative variables that were normally distributed, Student’s *t*-test was used; for those abnormally distributed, the Mann–Whitney test was used. We used the Kruskal–Wallis test for quantitative variables with abnormal distribution for groups larger than two. Furthermore, the Spearman coefficient was used to determine the correlation between quantitative variables. In order to evaluate the diagnostic utility of biomarkers, ROC was used. Then, an odds ratio (OR) was calculated and used. The most independent/affecting component was found using both univariate and multivariate regression analysis, and the 5% level of significance was applied to the findings.

## 3. Results

**Figure 2:**
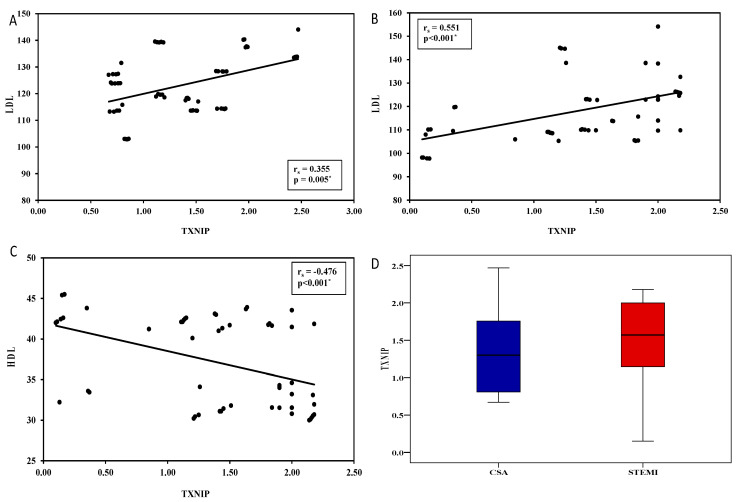
(**A**) Correlation between TXNIP and LDL in group II (**B**) Correlation between TXNIP and LDL in group III(**C**) Correlation between TXNIP and HDL in group III (**D**) Relation between CSA and CS according to TXNIP expression.

This study included a total of 60 patients with CSA (Group II) and 55 ACS patients (Group III) from the Department of Cardiology, Menoufia University Hospitals, Egypt, and 100 subjects with normal coronary angiography as a control group (Group I). All patients were diagnosed with angiography. The demographic, clinical and lab data are summarized in Table 1 and Table 2. All the groups were matched for age (*p* = 0.085) and gender (*p* = 0.3), as shown in Table 1. Patients with CAD had higher distributions of diabetes, hypertension, and smoking (*p* < 0.001; Table 1). Furthermore, patients with ACS had a higher percentage of two coronary vessel diseases (41.8%), WMAS (100%), and low EF than those with CSA (*p* < 0.001). Additionally, 27.3%, and 72.7% of patients with ACS presented with STEMI and NSTEMI on ECG (*p* < 0.001), respectively. On the contrary, 66.7% of patients with CSA had a previous history of ACS (*p* < 0.001; Table 1). As expected, patients with CAD had significantly higher levels of TC, LDLc TGs, VLDLC, and lower HDLc (*p* < 0.001) (mainly those with ACS (*p* < 0.001) and low Hb (*p* = 0.003), as shown in Table 2. RT-qPCR was applied to assess *PIK3C2A* and *TXNIP* expressions in the studied groups, and the results revealed an upregulation of *TXNIP* and downregulation of *PIK3C2A* in patients with CAD as compared to the control group (Group I) (*p* < 0.001; Table 2). It was found that *PIK3C2A* was significantly downregulated in patients with ACS compared to those with CSA (*p* < 0.001). However, the expressions of *TXNIP* revealed non-significant difference between ACS and CSA patients (*p* = 0.7; Table 2), except for STEMI (*p* = 0.045) Figure 2D. Moreover, the expression of TXNIP was significantly upregulated in STEMI-ACS patients as compared to non-STEMI ACS (*p* = 0.046) Table 3. Furthermore, it was upregulated in non-diabetic patients (*p* = 0.023), smokers (*p* = 0.036), patients with ACS, and those with hypertension (*p* = 0.005) and hypercholesterolemia (*p* = 0.001). CSA male (*p* = 0.01) hypertensive (*p* = 0.005) patients with hypercholesterolemia (*p* = 0.001) had significantly increased TXNIP expression. On the other hand, the expression of *PIK3C2A* was downregulated in smokers (*p* = 0.015), CSA patients with WMAS (*p* = 0.025), and in male (*p* = 0.001) smokers (*p* = 0.011)ACS patients (Table 3). However, the expression level of mRNAs *PIK3C2A* and *TXNIP* expression did not vary in CSA and ACS regarding the number of the affected coronary vessels (Table 3).

The diagnostic efficacy of *PIK3C2A* and *TXNIP* in patients with CAD was then determined by a ROC curve (Table 4). The most sensitive mRNA for discriminating ACS from control was *PIK3C2A* (0.981; *p* < 0.001; 98.18), followed by *TXNIP* (0.775; *p* < 0.001; 70.91). However, combined mRNAs (*PIK3C2A-TXNIP*) with a sensitivity of 98.18% and specificity of 83.33% at AUC 0.983, followed by *PIK3C2A* (0.892; *p* < 0.001; 81.82), distinguish ACS from CSA. On the other hand, the most sensitive mRNA for differentiating CSA from control is mixed mRNAs (*PIK3C2A–TXNIP*) with sensitivity of 95% (AUC; 0.963, *p* < 0.001), followed by *TXINP* (81.3; *p* < 0.001; 93.33), and finally, *PIK3C2A* (0.782; *p* < 0.001; 81.67), as shown in Table 4. Furthermore, mRNA *TXNIP* was positively correlated with T. cholesterol ((r = 0.3, *p* = 0.019) (r = 0.519, *p* < 0.0010) (not shown), LDLc ((r = 0.355, *p* = 0.005) (r = 0.551, *p* < 0.001))m among CSA and ACS, respectively, Figure 2B,C, and negatively correlated with HDLc in ACS (r = −0.476, *p* < 0.001)), Figure 2A. 

In addition, *PIK3C2A* (*p* = 0.00), 0.118 (0.031–0.445)) and smoking status (*p* = 0.034; 0.151 (0.026–0.866)) were independently related to ACS (Table 5). Furthermore, *PIK3C2A* (*p* < 0.013; 0.706 (0.614–0.812)), Hb (*p* = 0.013; 0.525 (0.317–0.871)), and TC (*p* = 0.04; 0.865 (0.784–0.955)) were significantly (*p* < 0.05) and independently related to the prognosis of CSA (Table 5). On the other hand, *PIK3C2A* (*p* = 0.002), 0.923 (0.877–0.971), *TXNIP* (*p* = 0.001; 2.809 (1.558–5.064)), and body weight (*p* = 0.033; 1.254 (1.018–1.544)) were independently associated with CSA (Table 5). 

## 4. Discussion

This study evaluated the potential role of *PIK3C2A* and *TXNIP* expressions that could be used as biomarkers for diagnosing and predicting the prognosis of CAD. The most serious form of CAD is ACS, a multifactorial disorder that endangers human health [25,26]. Despite significant advances in treatment, ACS, mainly, AMI, remains a global disease with high morbidity and mortality [8]. Our study showed significant downregulation of *PIK3C2A* in CAD patients compared to that in the control group. Moreover, *PIK3C2A* expression is lower in patients with ACS than those with CSA. Furthermore, combined mRNAs (*PIK3C2A + TXNIP*) followed by *PIK3C2A* are promising biomarkers for the diagnosis of ACS. *PIK3C2A* is a predictor of ACS development and prognosis of CSA. According to the previous literatures, *PIK3C2A* is a member of phosphoinositide 3-kinases (PI3Ks), which is a group of enzymes that control many signaling pathways by phosphorylating the inositol ring of phosphatidylinositol (PI) [14]. It is expressed in vascular endothelium, smooth muscle, and endothelial cells [14]. In line with our findings, a retrospective investigation found that the level of *PIK3C2A* gene expression was significantly lower in patients with AMI than in healthy people [27], indicating the potential role of low *PIK3C2A* expression as an independent variable in predicting the risk of AMI. Furthermore, it was reported that *PIK3C2A* expression was discovered to be negatively impacted by miR-206, which had an adverse influence on the angiogenesis process in vitro, and knockdown of miR-206 activated PIK3C2α, thus, promoting the angiogenic signal modulators Akt and eNOS [13].

Furthermore, in an established rat model of MI, Xiaoyu et al. 2021 investigated the molecular mechanism underlying the PI3K/Akt signaling pathway, miR-223-3p, and *PIK3C2A* potential role and provided new theories and concepts for effective prevention and treatment of coronary heart diseases [28]. A prior study revealed that *TXNIP* overexpression induced blood–brain barrier disruption in ischemic stroke and myocardial ischemia/reperfusion injuries [29]. In agreement with this study, our result demonstrated that *TXNIP* was overexpressed in patients with CAD compared to the control group. Mixed mRNAs (*PIK3C2A + TXNIP*) are a more sensitive biomarker in the diagnosis CSA followed by *TXNIP*, which, is a predictor of CSA. However, compared to CSA no significant association of *TXNIP* with ACS was observed except for those with STEMI. Additionally, Bedarida et al. (2018) discovered that endothelial *TXNIP* mediates arterial damage brought on by metabolic stress via an imbalance in oxidative stress and inflammation. It was demonstrated that the TRX-TXNIP system balance is critical for cardiomyocytes survival during ischemia [30]. The TRX-TXNIP system is disrupted in ischemic cardiomyopathy with reduced TRX and overexpressed *TXNIP*, but similar features are not evident in dilated cardiomyopathy [31].

In several different cell types, hypoxia induces the gene encoding TXNIP. However, an in vitro study contends that hypoxia causes a sharp decline in the expression of mRNA TXNIP. Therefore, hypoxia would regulate TXNIP expression in a biphasic manner [32].

In contrast, Zhang et al. (2017) showed that the *TXNIP* gene is upregulated in leukocytes in patients with unstable angina, but this phenomenon does not exist in patients with AMI [33,34]. This discrepancy could be attributed to the high smoking rate in their patients with AMI, which is considered a high-risk factor for AMI. Furthermore, CAD is influenced by various pathological processes and other elements that can alter the expression level of a single gene [33,34]. Moreover, Rong et al. (2020) concluded that increased *TXNIP* caused by demethylated cg19693031 may activate the nucleotide-binding oligomerization domain (NOD)-like receptor family, pyrin domain containing 3 (NLRP3) inflammasome, leading to a cascade of subsequent inflammatory reactions and promoting the activation of monocyte inflammation and formation of macrophages/foam cells, eventually leading to the occurrence of cardiovascular events [24,34]. These findings are consistent with a growing number of studies focusing on *TXNIP* to inhibit the ROS-TXNIP-NLRP3 pathway and attenuate myocardial ischemia-related heart damage [24,34,35]. Furthermore, only *TXNIP* was found to be differentially expressed between the ACS group and healthy controls, implying that *TXNIP* isoforms may have diverse functions in the development of ACS [36]. The carotid intima-media thickness (CIMT) in early diabetes and those with impaired glucose regulation was linked to enhanced TXNIP through the increased soluble vascular cell adhesion molecule-1 (sVCAM-1) that facilitates leukocyte adhesion to the endothelium, which is one of the first steps in the initiation of atherosclerosis [37]. These findings imply that TXNIP might serve as a prognostic indicator for the onset of vascular disease [37]. Therefore, therapies that target endothelial TXNIP may postpone the development of cardiovascular complications brought on by aging and its comorbidities, as well as endothelial dysfunction [38].

Smoking, hypertension, hyperlipidemia, inflammation caused by oxidative stress, apoptosis, vascular remodeling, plaque stress, blood-flow shear stress, and diabetes mellitus are all risk factors for ACS [39,40]. Moreover, Yao et al. (2021) hypothesized that inflammation and a family history of high cholesterol burden, which begins at a young age, are linked to CAD severity [41]. Similarly, our study revealed that hypercholesterolemia among those with CSA is an independent risk factor for the development of ACS. In addition, patients with hypercholesterolemia represent high levels of TXNIP expression. These combined findings indicate that *PIK3C2A* and *TXNIP* may play inflammatory roles in the development of CAD and suggest that *PIK3C2A* may be a novel therapeutic focus in CAD, which requires further studies with the follow-up of involved participants.

## 5. Conclusions

Our study deduced that dysregulation of mRNA *PIK3C2A* (downregulation) and *TXNIP* (upregulation) gene expressions among patients with CSA and those with ACS mainly STEMI patients may be helpful diagnostic biomarkers for CAD even more after the combination of these mRNAs, which could improve their diagnostic performance. mRNA *TXNIP* and *PIK3C2A* were independently related to CSA and ACS development, respectively. Thus, we recommend future studies to evaluate the therapeutic roles of *PIK3C2A* and *TXNIP* to reduce the incidence of ACS development.

## Figures and Tables

**Table 1:** Comparison of the involved groups based upon demographic and clinical parameters.

	Group I (n = 100)	Group II (n = 60)	Group III (n = 55)	Test of Sig.	*p*	Significance between Groups		
						I vs. II	I vs. III	II vs. III
Gender								
Male	76 (76%)	45 (75%)	47 (85.5%)	χ^2^ = 2.337	0.3	NS	NS	NS
Female	24 (24%)	15 (25%)	8 (14.5%)					
Age (years)								
Mean ± SD	54.6 ± 6	53.7 ± 7.7	56.7 ± 9.4	F = 2.494	0.9	NS	NS	NS
BMI								
Mean ± SD	28.6 ± 3.6	29.3 ± 4.5	30.4 ± 3.5	F = 3.785 *	0.02 *	0.488	0.018 *	0.308
Smoking No Smoker Ex-smoker	69 (69%) 21 (21%) 10 (10%)	20 (33.3%) 20 (33.3%) 20 (33.3%)	13 (23.6%) 25 (45.5%) 17 (30.9%)	χ^2^ = 38.092	<0.001 *	<0.001 *	<0.001 *	0.355
DM	10 (10%)	20 (33.3%)	18 (32.7%)	χ^2^ = 16.385 *	<0.001 *	<0.001 *	<0.001 *	0.945
HTN	10 (10%)	25 (41.7%)	30 (54.5%)	χ^2^ = 38.540 *	<0.001 *	<0.001 *	<0.001 *	0.167
Previous MI	0 (0%)	40 (66.7%)	5 (9.1%)	χ^2^ = 106.968 *	<0.001 *	<0.001 *	^FE^*p* = 0.005 *	<0.001 *
CA								
Normal	100 (100%)	0 (0%)	0 (0%)	χ^2^ = 339.658 *	^MC^*p* < 0.001 *	^MC^*p* < 0.001 *	<0.001 *	^MC^*p* < 0.001 *
1VD	0 (0%)	10 (16.7%)	22 (40%)					
2VD	0 (0%)	10 (16.7%)	23 (41.8%)					
3VD	0 (0%)	25 (41.7%)	10 (18.2%)					
Presentation								
CSA	0 (0%)	60 (100%)	0 (0%)	χ^2^ = 430.0 *	<0.001 *	<0.001 *	<0.001 *	<0.001 *
NSTEMI	0 (0%)	0 (0%)	15 (27.3%)					
STEMI	0 (0%)	0 (0%)	40 (72.7%)					
EF								
Mean ± SD	61.2 ± 1	56.5 ± 11.4	50.9 ± 7.2	F = 36.2 *	<0.001 *	<0.001 *	<0.001 *	<0.001 *
WMAS								
No	100 (100%)	10 (16.7%)	0 (0%)	χ^2^ = 181.65 *	<0.001 *	<0.001 *	<0.001 *	^FE^*p* = 0.001 *
Yes	0 (0%)	50 (83.3%)	55 (100%)					
Systolic BP								
Mean ± SD	127 ± 7	127.5 ± 20.2	126.5 ± 14	F = 0.07	0.9	NS	NS	NS
Diastolic BP								
Mean ± SD	85.8 ± 4.8	84.2 ± 12.1	86.5 ± 7.6	F = 1.2	0.3	NS	NS	NS
Pulse								
Mean ± SD	91.1 ± 3.3	76.4 ± 10.3	86.5 ± 3.3	F = 108.5 *	<0.001 *	<0.001 *	<0.001 *	<0.001 *

BMI: body mass index, DM: diabetes mellitus, HTN: hypertension, MI: myocardial infarction, CA: coronary artery, VD: vessels disease, NSTEMI: non ST elevation myocardial infarction, STEMI: ST elevation myocardial infarction, EF: ejection fraction, WMAS: wall motion abnormalities, χ^2^: Chi-square test, F: F for ANOVA test, *p*: *p* value for association between different parameters. *: Statistically significant at *p* ≤ 0.05.

**Table 2:** Comparison between the studied groups regarding biochemical parameters.

	Group I (n = 100)	Group II (n = 60)	Group III (n = 55)	Test of Sig.	*p*	Significance between Groups		
						I vs. II	I vs. III	II vs. III
T. cholesterol (mg/dL)								
Mean ± SD	160.7 ± 6.4	185.9 ± 10.7	181.5 ± 8.6	F = 207.7 *	<0.001 *	<0.001 *	<0.001 *	0.013 *
LDLc (mg/dL)								
Mean ± SD	89.2 ± 6.4	123 ± 11	118.6 ± 13.6	F = 272.2 *	<0.001 *	<0.001 *	<0.001 *	0.048 *
HDLc (mg/dL)								
Mean ± SD	51.1 ± 1.9	39.9 ± 5.2	37.1 ± 5.5	F = 256.3 *	<0.001 *	<0.001 *	<0.001 *	0.001 *
TGS (mg/dL)								
Mean ± SD	102 ± 7.3	114.9 ± 15.4	129 ± 18.8	F = 72.2	<0.001 *	<0.001 *	<0.001 *	0.001 *
VLDLc (mg/dL)								
Mean ± SD	20.4 ± 1.5	23 ±3.1	25.8 ± 3.8	F = 72.2	<0.001 *	<0.001 *	<0.001 *	0.001 *
Hypercholesterolemia Yes No	0 (0%) 100 (100%)	35 (58.3%) 25 (41.7%)	28 (50.9%) 27 (49.1%)	χ^2^ = 78.3	<0.001 *	<0.001 *	<0.001 *	0.424
Troponin Negative Positive	100 (100%) 0 (0%)	60 (100%) 0 (0%)	0 (0%) 55 (100%)	χ^2^ = 215.0 *	<0.001 *	–	<0.001 *	<0.001 *
HB (gm/dL)								
Mean ± SD	14.7 ± 0.8	13.8 ± 1	12.9 ± 2.2	F = 29.99 *	<0.001 *	<0.001 *	<0.001 *	0.003 *
PIK3C2A								
Mean ± SD	0.793 ± 0.6	0.218 ± 0.1	0.054 ± 0.03	H = 122.4 *	<0.001 *	<0.001 *	<0.001 *	<0.001 *
Median (Min.–Max.)	0.675 (0.08–1.77)	0.26 (0.04–0.38)	0.056 (0.01–0.093)					
TXNIP								
Mean ± SD	0.8 ± 0.3	1.3 ± 0.5	1.4 ± 0.7	H = 55.6 *	<0.001 *	<0.001 *	<0.001 *	0.700
Median (Min.–Max.)	0.6 (0.4–1.2)	1.3 (0.7–2.5)	1.5 (0.1–2.2)					

T. cholesterol: total cholesterol, LDLc: low density lipoprotein, HDLc: high density lipoproteins, TGS: triglycerides, Hb: hemoglobin concentration, WBCs: white blood cells; PLT: platelet, PIK3C2A: phosphoinositide 3-kinase, TXNIP: thioredoxin-interacting protein, χ^2^: Chi-square test, F: F for ANOVA test, H: H for Kruskal–Walli’s test, *p*: *p* value for association between different parameters. *: Statistically significant at *p* ≤ 0.05.

**Table 3:** Relation between mRNA (PIK3C2A, TXNIP) gene expression level and different parameters in CSA and ACS patients.

		CSA			ACS	
	N	PIK3C2A	TXNIP	N	PIK3C2A	TXNIP
		Median (Min.–Max.)	Median (Min.–Max.)		Median (Min.–Max.)	Median (Min.–Max.)
Gender						
Male	45	0.26 (0.04–0.38)	1.42 (0.67–2.47)	47	0.05 (0.01–0.09)	1.41 (0.10–2.18)
Female	15	0.26 (0.05–0.38)	0.77 (0.68–1.79)	8	0.08 (0.07–0.09)	1.71 (1.42–2.18)
U(*p*)		320.50 (0.771)	187.0 * (0.010 *)		52.0 * (0.001 *)	122.50 (0.119)
DM						
No	40	0.26 (0.04–0.38)	1.30 (0.67–1.99)	37	0.05 (0.01–0.09)	1.81 (0.15–2.18)
Yes	20	0.18 (0.04–0.37)	1.45 (0.68–2.47)	18	0.06 (0.01–0.09)	1.39 (0.10–2.18)
U(*p*)		316.0 (0.186)	320.50 (0.213)		310.50 (0.686)	206.50 * (0.023 *)
HTN						
No	35	0.25 (0.04–0.38)	1.15 (0.67–1.78)	25	0.05 (0.01–0.09)	1.25 (0.10–2.18)
Yes	25	0.27 (0.04–0.38)	1.75 (0.69–2.47)	30	0.07 (0.01–0.09)	1.90 (0.15–2.18)
U(*p*)		434.0 (0.958)	252.0 * (0.005 *)		268.0 (0.070)	208.0 *(0.005 *)
Smoking						
No	20	0.21 (0.04–0.38)	1.24 (0.68–1.99)	13	0.08 (0.05–0.09)	1.43 (0.10–2.18)
Smoker	20	0.21 (0.04–0.38)	1.33 (0.67–1.78)	25	0.05 (0.01–0.09)	1.82 (0.85–2.18)
Ex-smoker	20	0.28 (0.16–0.38)	1.30 (0.82–2.47)	17	0.03 (0.01–0.08)	1.25 (0.15–2.00)
H(*p*)		8.433 (0.015 *)	1.049 (0.592)		8.992 (0.011 *)	6.647 (0.036 *)
Presentation						
CSA	60	0.26 (0.04–0.38)	1.30 (0.67–2.47)	0	–	–
NSTEMI	0	–	–	15	0.06 (0.02–0.08)	1.22 (0.10–2.00)
STEMI	0	–	–	40	0.06 (0.01–0.09)	1.57 (0.15–2.18)
CHEST PAIN	0	–	–	0	–	–
H(*p*)		–	–		259.0 (0.438)	194.50 * (0.046 *)
WMAS						
No	10	0.28 (0.19–0.37)	1.65 (0.82–2.47)	0	–	–
Yes	50	0.24 (0.04–0.38)	1.30 (0.67–1.99)	55	0.06 (0.01–0.09)	1.45 (0.10–2.18)
U(*p*)		137.50 (0.025 *)	175.0 (0.137)		–	–
Hypercholesterolemia						
Hypercholesterolemia	35	0.27 (0.04–0.38)	1.71 (0.69–2.47)	28	0.06 (0.01–0.09)	1.90 (0.13–2.18)
No-hypercholesterolemia	25	0.25 (0.04–0.28)	0.85 (0.67–1.52)	27	0.06 (0.01–0.09)	1.20 (0.10–2.18)
U(*p*)		351.50 (0.655)	185.50 (0.001 *)		351.50 (0.655)	185.50 (0.001 *)
Troponin						
Negative	60	0.26 (0.04–0.38)	1.30 (0.67–2.47)	0	–	–
Positive	–	–	–	55	0.06 (0.01–0.09)	1.45 (0.10–2.18)
U(*p*)		–	–		–	–
CA						
MVD	25	0.26 (0.04–0.38)	1.50 (0.68–2.47)	10	0.05 (0.02–0.08)	1.58 (1.20–2.00)
1VD	25	0.24 (0.04–0.38)	1.13 (0.67–1.99)	22	0.06 (0.04–0.09)	1.57 (0.15–2.18)
2VD	10	0.27 (0.05–0.38)	1.24 (0.69–1.79)	23	0.06 (0.01–0.09)	1.43 (0.10–2.18)
H(*p*)		2.010 (0.366)	5.475 (0.065)		2.055 (0.358)	0.171 (0.918)

DM: diabetes mellitus, HTN: hypertension, CA: coronary artery, VD: vessels disease, NSTEMI: non ST elevation myocardial infarction, STEMI: ST elevation myocardial infarction, EF: ejection fraction, WMAS: wall motion abnormalities, U: Mann–Whitney test, H: H for Kruskal–Wallis test. *p*: *p* value for comparison between the studied categories. *: Statistically significant at *p* ≤ 0.05.

**Table 4:** Analytical performance of mRNAs *PIK3C2A* and *TXNIP* to distinguish different studied groups.

	AUC	*p*	95%CI	Cut off	Sensitivity	Specificity	PV	NPV
Acute coronary syndrome from control								
PIK3C2A	0.981	<0.001 *	0.966–0.996	≤0.091	98.18	87.0	80.6	98.9
TXNIP	0.775	<0.001 *	0.673–0.876	>1.195	70.91	89.0	78.0	84.8
Acute coronary syndrome from chronic stable angina								
PIK3C2A	0.892	<0.001 *	0.828–0.957	0.081	81.82	83.33	81.8	83.3
TXNIP	0.572	0.184	0.464–0.680	>1.19	70.91	48.33	55.7	64.4
PIK3C2A + TXNIP	0.893	<0.001 *	0.829–0.957	98.18	83.33	84.4	98.0	
Chronic stable angina from control								
PIK3C2A	0.782	<0.001 *	0.708–0.856	≤0.28	81.67	73.0	64.5	86.9
TXNIP	0.813	<0.001 *	0.747–0.879	>0.7	93.33	58.0	57.1	93.5
PIK3C2A + TXNIP	0.963	<0.001 *	0.939–0.987	95.0	80.0	74.0	96.4	

AUC: area under a curve; *p* value: probability value; CI: confidence intervals; NPV: negative predictive value; PPV: positive predictive value. *: Statistically significant at *p* ≤ 0.05.

**Table 5:** Univariate and multivariate logistic regression analysis for the parameters affecting studied groups.

Chronic Stable Angina Patients (n = 60) from Controls (n = 100).				
	**Univariate**		**^#^** **Multivariate**	
	***p***	**OR (95%CI)**	***p***	**OR (95%CI)**
PIK3C2A ^@1^	<0.001 *	0.958 (0.941–0.976)	<0.001 *	0.994 (0.991–0.997)
TXNIP ^@2^	<0.001 *	1.362 (1.228–1.510)	<0.001 *	2.635 (1.729–4.016)
Body weight	<0.001 *	1.146 (1.084–1.211)	<0.001 *	1.187 (1.085–1.299)
acute coronary syndrome patients (n = 55) from chronic stable angina patients (n = 60).				
	***p***	**OR (95%CI)**	***p***	**OR (95%CI)**
PIK3C2A	<0.001	0.728 (0.639–0.829)	<0.001 *	0.706 (0.614–0.812)
Hb	0.012 *	0.733 (0.575–0.935)	0.013 *	0.525 (0.317–0.871)
T. Cholesterol	0.019 *	0.954 (0.917–0.992)	0.004 *	0.865 (0.784–0.955)
acute coronary syndrome patients (n = 55) from controls (n = 100).				
	***p***	**OR (95%CI)**	***p***	**OR (95%CI)**
DM	0.001 *	4.378 (1.848–10.374)	0.131	0.248 (0.041–1.515)
Smoking	<0.001 *	7.191 (3.388–15.265	0.034 *	6.623 (1.155–37.971)
PIK3C2A^@1^	<0.001 *	0.175 (0.056–0.462)	0.002 *	0.118 (0.031–0.445

TXNIP: thioredoxin-interacting protein; PIK3C2A: phosphoinositide 3-kinase, Hb: hemoglobin concentration, T. Cholesterol: total cholesterol; DM: diabetes mellitus, CI: confidence interval, *p*: *p* value for association between different parameters. *: Statistically significant at *p* ≤ 0.05. ^@1^: for every 0. 01. ^@2^: for every 0.

## Data Availability

All the information and materials are accurate and adhere to professional standards. Although not publicly accessible, the datasets created and/or used in the current work are available upon reasonable request from the corresponding author.
